# DNA Binding and Photocleavage Studies of Cobalt(III) Ethylenediamine Pyridine Complexes: ${\text{[}\text{Co}{\text{(}\text{en}\text{)}}_{\text{2}}{\text{(}\text{py}\text{)}}_{\text{2}}\text{]}}^{\text{3}\text{+}}$ and ${\text{[}\text{Co}{\text{(}\text{en}\text{)}}_{\text{2}}{\text{(}\text{mepy}\text{)}}_{\text{2}}\text{]}}^{\text{3}\text{+}}$

**DOI:** 10.1155/2008/275084

**Published:** 2007-10-01

**Authors:** Penumaka Nagababu, D. Aravind Kumar, Kotha Laxma Reddy, K. Ashwini Kumar, Md. B. Mustafa, Mynam Shilpa, S. Satyanarayana

**Affiliations:** ^1^Department of Chemistry, Faculty of Science, Osmania University, Hyderabad 500 007, Andhra Pradesh, India; ^2^Indian Institute of Chemical Technology, Taranaka, Hyderabad 500 007, India

## Abstract

Two novel cobalt(III) pyridine complexes **(1)** ${\left[\text{Co}{\left(\text{en}\right)}_{2}{\left(\text{py}\right)}_{2}\right]}^{3+}$ and **(2)** ${\left[\text{Co}{\left(\text{en}\right)}_{2}{\left(\text{mepy}\right)}_{2}\right]}^{3+}$ (en=ethylenediamine, py=pyridine, and mepy=methylpyridine) have been synthesized and characterized. The interaction of these complexes with calf thymus DNA was investigated by absorption, emission spectroscopy, viscosity measurements, DNA melting, and DNA photocleavage. Results suggest that the two complexes bind to DNA via groove mode and complex **2** binds more strongly to CT DNA than complex **1**. Moreover, these Co(III) complexes have been found to promote the photocleavage of plasmid DNA pBR322 under irradiation at 365 nm, cytotoxicity results of complexes are also showing anticancer activity.

## 1. INTRODUCTION

The interaction of transition metal polypyridyl complexes with DNA has received a great deal of attention during the past decade [1–3]. Many complexes have been synthesized. These complexes can bind to DNA in noncovalent modes such as electrostatic, intercalative, and groove binding [4, 5]. The cationic metal complexes possessing planar aromatic ligands may bind to DNA by intercalation which involves stacking of the planar ligand in between adjacent base pairs of the DNA duplex [6–9]. In the early 1980s, Barton demonstrated that tris phenanthroline complexes of ruthenium (II) display enantiomeric selectivity in binding to DNA, which can be served as spectroscopic probes in solution to distinguish right- and left-handed DNA, helices [10]. Then they found that tris (phenanthroline) complexes of cobalt(III) could cleave DNA when irradiated at 254 nm. Furthermore, they conducted the cleavage reactions by using high stereo specificity of tris (diphenyl penanthroline) (DIP) metal isomers. The cleavage reaction is also stereo specific. These findings underscore the importance of an intimate association of the metal ion with the duplex. The high level of recognition of DNA conformation by these chiral inorganic complexes suggested the powerful application of stereo specificity in DNA drug design [11]. According to [12], *cis*- and *trans*-[PtCl_2_(pyridine)_2_] complexes show anticancer activity and inhibit DNA synthesis, implying a role for DNA binding in their mechanism of action, and *cis* complex implies more binding with CT DNA than trans complex.

A series of dichloro(ethylenediamine)-type platinum complexes bearing ester-, amide-, and ether-bonded alkyl straight chains were prepared as a model for the prodrug of *cis*-diamminedichloroplatinum [13] and the cytotoxic activity of the complexes against the S-180 cell line was investigated. Schonenberger et al. presented antitumor active (1,2-diphenylethylenediamine)-platinum (II) complex compounds [14]. Ring-substituted diaqua(1,2-diphenylethylenediamine) platinum(II) sulfate was prepared [15] and mode of binding to the DNA was studied. A series of isomeric[1,2-*bis* (difluorophenyl) ethylenediamine] dichloroplatinum (II) complexes and *cis*-platin were tested on the P388 leukemia and on the murine mammary carcinoma for evaluating antineoplastic activity against breast cancer in vivo [16]. The activity of 1,2-*bis*(2,6-difluoro-3-hydroxy-phenyl)ethylenediamine] platinum(II) complexes against breast cancer was investigated in [17].

Our group has synthesized some Ruthenium(II) and Cobalt(III) ethylenediamine mixed-polypyridyl complexes, which bind to DNA through an intercalative and groove mode and promote cleavage of plasmid pBR 322 DNA [18–21]. Herein we chose to concentrate on the cobalt(III)ethylenediamine complexes, because they have same interesting characteristics of metallointercalation. In this paper, we are reporting the synthesis and characterization of the complexes 1 and 2 in which 2 possesses a greater binding affinity and their DNA-binding properties are revealed by electronic absorption, emission spectra, viscosity measurement, and DNA melting curve. The photochemical DNA cleavage of the complexes is also demonstrated. These studies are necessary for further comprehension of binding of transition metal complexes to DNA. The cytotoxicity studies of 1 and 2 complexes were discussed in this paper.

## 2. EXPERIMENTAL

*Materials*. All materials were purchased and used without further purification unless otherwise noted. Pyridine, ethylenediamine, and CT DNA were purchased from *Aldrich*. All the experiments involving interaction of the complexes with DNA were carried out in BPE buffer (5 mM Tris-HCl, 50 mM NaCl, pH 7.0). A solution of calf thymus DNA in the buffer gave a ratio of UV absorbance at 260 and 280 nm of about 1.90 indicating that the DNA was sufficiently free of protein [22]. The DNA concentration per nucleotide was determined by absorption spectroscopy using the molar absorption coefficient (6600 M^−1^cm^−1^) at 260 nm [23].

## 3. SYNTHESIS OF COMPLEXES

### 3.1. [Co(en)_2_(py)_2_]^3+^

A mixture of *cis*-[Co(en)_2_Cl_2_]Cl (1.43 g) was prepared by the procedure available in the literature (see [24]). Complexes 1 and 2 were prepared by literature methods [25–27] as follows. A mixture of *trans*-[Co(en)_2_Cl_2_]Cl {4.28 g, 0.015 mol} and pyridine {2.7 g, 0.015 mol} taken in distilled H_2_O (20 cm^3^) was heated at 100°C for 30 minutes. A saturated NaBr solution was added to the cooled solution, which was kept overnight. After filtration to remove [Co(en)_3_]Br_3_, Me_2_CO (200 cm^3^) was added, resulting in precipitation of a mixture of [Co(en)_2_(py)_2_]Br_3_ and [Co(en)(py)_4_]Br_3_. This was dissolved in H_2_O and the complex [Co(en)_2_(py)_2_]Br_3_ was reprecipitated by addition of EtOH. [Co(en)_2_(Mepy)_2_]Br_3_, (2) was prepared similarly using methylpyridine. UV/Vis: 361,470 and 618 nm Isosbestic points: 438 and 576 nm IR: 1457 (C=C), 1578 (C=N), 469 (Co−N (en)), 578 cm^−1^ (Co−N (L)). Formula: Co N_6_H_26_C_14_Br_3_Anal. Calc. H 4.54, C 29.14, N 14.56 found: H 4.01, C 29.0, N 14.02. ^1^H-NMR (D_2_O), 3.1,(dd, 2CH_2_ (en)_2_, 2.55(m,2CH_2_(en)_2_, 7.69 (d, 2H), 8.210 (d, 2H), 7.990(t, 1H).

### 3.2. [Co(en)_2_(mepy)_2_]^3+^

UV/Vis: 312, 447 and 617 nm, Isosbestic points: 449 and 578 nm IR: 1448 (C=C), 1577 (C=N), 467 (Co−N (en)), 555 cm^−1^ (Co−N (L)). Formula: Co N_6_H_30_C_16_Br_3_, Anal. Calc. H 5.00, C 31.76 N 13.89 found: H 4.8 C 30.56 N 12.18. ^1^H-NMR (D_2_O), 2.7,(dd, 2CH_2_ (en)_2_, 2.89(m, 2CH_2_(en)_2_, 7.412(d, 2H), 8.036(d,2H) 4.412(s, 3H).

### 3.3. Physical measurements

UV-Visible spectra were recorded on *Elico Bio*-spectrophotometer model *BL198*, emission spectra were recorded on a *Shimadzu Rf-2000* luminescence spectrometer at room temperature. IR spectra were recorded, in KBr phase on *Perkin-Elmer FTIR-1605* spectrophotometer; ^1^H-NMR spectra were measured on a *Varian XL-300* MHz spectrometer with D_2_O as a solvent at room temperature and tetramethylsilane (TMS) as the internal standard, Microanalyses (C, H, N) were carried out on a *Perkin-Elmer* 240 elemental analyzer.

For the absorption spectra titrations were carried out at room temperature to determine the binding affinity between DNA and complex. Initially, 3000 *μ*L solutions of the blank buffer and the cobalt complex sample (20 *μ*M) were placed in the reference and sample cuvettes (1 cm path length), respectively, and then first spectrum was recorded in the range of 200–600 nm. During the titration, aliquot (1–10 *μ*L) of buffered DNA solution (concentration of ∼5 to 10 mM in base pairs) was added to each cuvette to eliminate the absorbance of DNA itself, and the solutions were mixed for ∼5 minutes, the absorption spectra were recorded. The titration processes were repeated until there was no change in the spectra indicating that binding saturation had been achieved. The changes in the metal complex concentration due to dilution at the end of each titration were negligible. The cobalt (III) complexes on other hand, showed additional MLCT bands between 400–500 nm [28].

Emission measurements were carried out by using a HitachiF 4500 Fluorescence Spectrometer. Tris-buffer was used as a blank to make preliminary adjustments. The excitation wavelength was fixed and the emission range was adjusted before measurements. All measurements were made at 25° in a thermostated cuvette holder with 5 nm entrance slit and 5 nm exit slit. Emission titration experiments were performed at a fixed metal complex concentration (20 *μ*M) to which increments of a stock DNA solution (0–160 *μ*M) containing the same concentration of the metal complexes were added. The emission enhancement factors were measured by comparing the intensities at 559 nm in the absence and presence of CT DNA.

Viscosity experiments were carried out using an Ostwald viscometer maintained at a constant temperature 30.0±0.1° in a thermostatic water bath. Calf thymus DNA samples, approximately 200 base pairs in average length, were prepared by sonicating in order to minimize complexities arising from DNA flexibility [29]. Data were presented as ${\left(\eta /\eta_{0}\right)}^{1/3}$ versus the concentration of Co(III) complexes, where η is the viscosity of DNA in presence of complexes and $\eta_{0}$ is the viscosity of DNA alone. Viscosity values were calculated from the observed flow time of DNA-containing solution (t>100 seconds) corrected for flow time of buffer alone ($t_{0}$), $\eta =t-t_{0}$ [30]. The DNA melting experiments were done by controlling the temperature of the sample cell with a *Shimadzu* circulating bath while monitoring the absorbance at 260 nm.

Thermal denaturation studies were carried out with a *Elico Bio*-spectrophotometer model *BL198*, equipped with temperature-controlling programmer (±0.1°C). The absorbance at 260 nm was continuously monitored for solutions of CT-DNA (100 *μ*M) in the absence and presence of the cobalt(III) complex (10 *μ*M). The temperature of the solution was increased by 1°C min^−1^. For the gel electrophoresis experiments, super coiled pBR322 DNA (100 *μ*M) was treated with Co(III) complexes in pH = 7.2, and the solutions were incubated for 1 hour h in the dark. The samples were analyzed by electrophoresis for 2.5 hours at 40 V on a 0.8% agarose gel in buffer, pH 7.2. The gel was stained with 1 *μ*g/ml ethidiumbromide and then photographed under UV light.

### 3.4. Spectroscopic characterization

**Figure 1:**
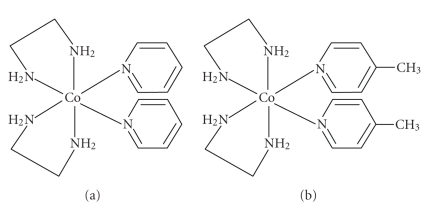
Molecular structure of complexes.

Molecular structures of the complexes are given in Figure 1. The IR spectral data for the complexes are given. The complexes clearly exhibit a band at 1458 cm^−1^and 1578–1590 cm^−1^ corresponding to C=C and C=N of the ring, respectively. A band at around 589 cm^−1^ and 590 cm^−1^ corresponding to Co−N(en) and Co−N of NH_2_(en) bending exhibits around 1650 cm^−1^. In the ^1^H-NMR spectra of the Co(III) complexes, the peaks due to various protons of pyridine shifted downfield compared to the free ligand suggesting complexation. As expected the signal for pyridine appeared in the range between 6.5 to 9.2, CH_2_ of ethylenediamine gave peaks at 3.1 (br, 4 H, CH_2_(en)).

### 3.5. Cell viability MTT assay 

All cell culture reagents and media were purchased from Sigma-Aldrich and used without further purification unless otherwise noted. Cytotoxicity assay were performed using Chinese hamster ovarian (CHO) in order to assess the cancer chemotherapeutic potential of the cells. Cells were grown as monolayers in Eagle's minimum essential medium, supplemented with 2 mM L-glutamine and Earle's balanced salt solution, containing 1.5 g dm^−3^, sodium bicarbonate, 0.1 mM nonessential amino acids, 1.0 mM sodium pyruvate, 100 cm^−3^ penicillin, and 100 *μ*gcm^−3^ streptomycin supplemented to contain 10% (v/v) foetal bovine serum. All cells were grown at 37°C in a humidified atmosphere, in the presence of 5% CO_2_, and were in the exponential phase of growth at the time of assay. Cytotoxicity was assessed using MTT assay. Cells (100 *μ*L) were seeded at a density of $5\times 10^{4}$ cells cm^−3^ into sterile 96 well flat-bottomed plates (Falcon, Plastics, Becton, Dickinson) and grown in 5% CO_2_ at 37°C. Test compounds were dissolved in culture media. Each drug solution (100 *μ*L) was added to replicate wells in the concentration range of 0.1–100 *μ*M and incubated for 72 hours. A miniaturized viability assay using 3-(4,5-dimethylthiazol-2-yl)-2,5-diphenyl tetrazolium bromide (MTT) was carried out according to method described by Mosmann [31]. The IC_50_ value, defined as the drug concentration causing a 50% reduction in cellular viability was calculated for each drug. Each assay was carried out using five replicates and repeated on at least three separate occasions. Viability was calculated as a percentage of solvent-treated control cells, and expressed as a percentage of the control. The significance of any reduction in cellular viability was determined using one-way ANOVA (analysis of variance). A probability of .05 or less was deemed statistically significant.

## 4. RESULTS AND DISCUSSION

### 4.1. Absorption spectral studies

**Figure 2:**
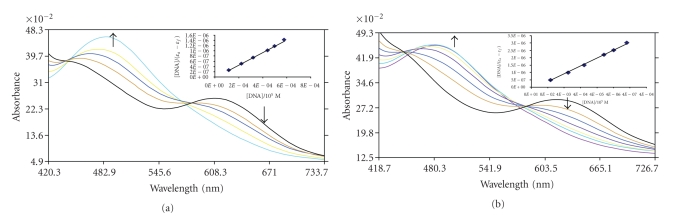
Absorption spectra of complexes: (a) complex 1, (b) complex 2, in tris-HCl buffer. Upon addition of CT DNA to complex absorption decreases [Co] = 10 *μ*M; [DNA] = 0–126 *μ*M. Insert: plots of $\text{(}\epsilon_{\text{a}}-\epsilon_{\text{f}}\text{)/(}\epsilon_{\text{b}}-\epsilon_{\text{f}}\text{)}$ versus [DNA] for the titration of DNA with Co(III) complexes. Isosbestic points at 438, 576 for complex 1. Isosbestic points at 449, 578 for complex 2.

Absorption titration experiments of Co(III) complexes in buffer were performed by using fixed cobalt complex concentration to which increments of the DNA stock solution were added. The calf thymus DNA was added to a ratio of 8:1 [DNA]/[Co]. Cobalt solutions were allowed to incubate for 10 minutes before the absorption spectra were recorded (see Figures 2(a) and 2(b)). As the DNA concentration is increased, the MLTC transition bands of complex at 618 nm exhibit hypochromism and as well as an insignificant bathochromism, showing isosbestic points at 438, 576 and 449, 578 complexes 1 and 2, respectively. Based on the observations of complexes, we presume that there are some interactions between complexes and DNA. To know quantitatively the binding strength of the complexes, the intrinsic binding constant $K_{\text{b}}$ of the complexes with CT-DNA were obtained by monitoring the changes in absorbance at 618 and 617 nm for complexes (1 and 2, resp.) with increasing concentration of DNA using the following function equation [32], which has been applied to describe the binding of high-affinity complexes to DNA assuming noncooperative binding to discrete sites:

(1) $$\left[\text{DNA}]/(\epsilon_{\text{a}}-\epsilon_{\text{f}})=[\text{DNA}]/(\epsilon_{\text{b}}-\epsilon_{\text{f}})+1/(\text{K}(\epsilon_{\text{b}}-\epsilon_{\text{f}})\right),$$

where [DNA] is the concentration of DNA in base pairs, the apparent absorption coefficients $\epsilon_{\text{a}}$, $\epsilon_{\text{f}}$ and $K_{\text{b}}$ correspond to $A_{\text{obs}}$/[Co], the extinction coefficient for cobalt complexes in the free and fully bound form, respectively. In plots [DNA]/$\left(\epsilon_{\text{a}}-\epsilon_{\text{f}}\right)$ versus [DNA]. K is given by the ratio of slope to intercept. Intrinsic binding constants K obtained about $2.7\pm 0.2\times 10^{3}$ and $3.5\pm 0.2\times 10^{3}$ of complexes 1 and 2, respectively, from the decay of the absorbance. The binding constants indicate that complex 2 binds more strongly than 1 to CT DNA.

### 4.2. Emission studies

**Figure 3:**
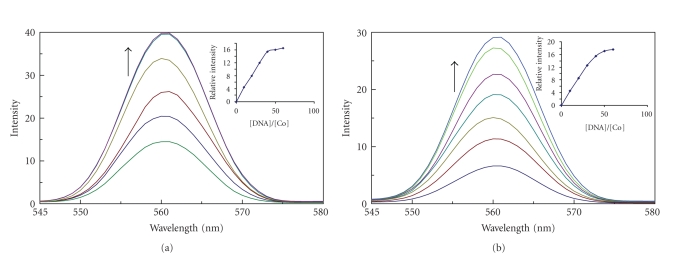
Fluorescence emission spectra of complexes: (a) complex 1, (b) complex 2 in tris-HCl buffer. Fluorescence intensity increases upon increasing CT DNA concentrations (5 *μ*l, 10 *μ*l, 15 *μ*l, 20 *μ*l, …). Insert: plots of relative emission intensity versus [DNA]/[Co].

In the absence of DNA, complexes can emit luminescence in Tris buffer with emission maximum appearing at 562 nm. Upon addition of CT DNA (= Calf thymus DNA), the emission intensities of the complexes increase when compared to the intensity of complexes alone shown in Figures 3(a) and 3(b). This implies that complexes can strongly interact with DNA and be protected by DNA efficiently, since the hydrophobic environment inside the DNA helix reduces the accessibility of solvent water molecules to the duplex and the complexes mobility is restricted at the binding site, lead to decrease the vibrational modes of relaxation.

**Figure 4:**
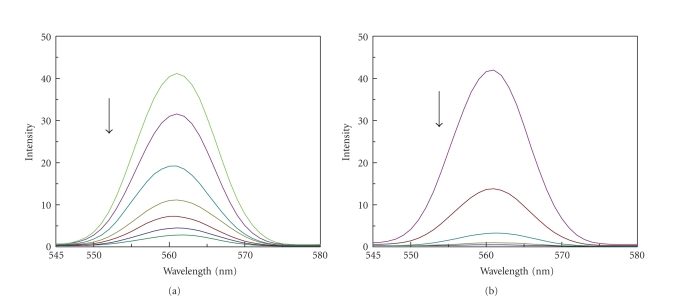
Fluorescence quenching curves of DNA + complex by ferrocyanide: (a) complex 1 + DNA; (b) complex 2 + DNA.

**Figure 5:**
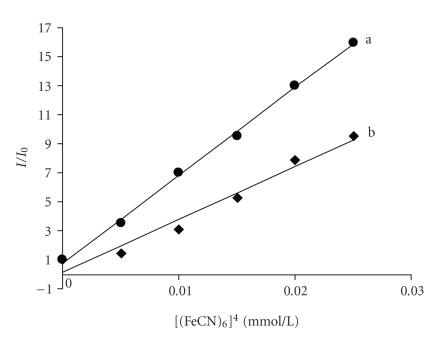
Quenching of fluorescence emission of Co(III) complex + DNA with Ferro cyanide: (a) complex1 + DNA; (b) complex 2 + DNA.

This observation is further supported by the fluorescence quenching experiments using [Fe(CN)_6_]^4−^ as quencher. The ion [Fe(CN)_6_]^4−^ has been shown to be able to distinguish differentially bound Co(III) species and positively charged free complex ions should be readily quenched by [Fe(CN)_6_]^4−^. The complexes bound to DNA can be protected from the quencher, because highly negatively charged [Fe(CN)_6_]^4−^ would be repelled by the negative DNA phosphate backbone, hindering quenching of the emission of the bound complex. The method essentially consists of titrating a given amount of DNA-metal complexes with increasing the concentration of [Fe(CN)_6_]^4−^ and measuring the change in fluorescence intensity (see Figure 4). The ferro-cyanide quenching curves for these complexes in the presence and absence of CT DNA are shown in Figure 5. Obviously, complex 2 inserts into DNA much deeper than 1. The absorption and fluorescence spectroscopy studies determine the binding of complexes with DNA.

### 4.3. Viscosity studies

**Figure 6:**
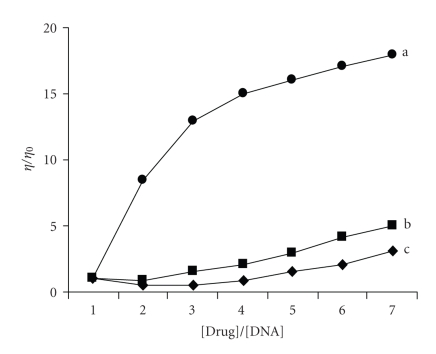
Effect of increasing amount of complexes on the relative viscosities of CT DNA at $\text{25}\pm \text{0}{\text{.1}}^{\circ }$: (a) EtBr, (b) complex 2, (c) complex 1.

Mode of interaction between the metal complexes and DNA was clarified by viscosity measurements. Optical photophysical probes are necessary, but not sufficient to support a binding model. Hydrodynamic measurements are sensitive to length change (i.e., viscosity and sedimentation) are regarded as the least ambiguous and the most critical tests of binding in solution in the absence of crystallographic structural data [33]. A classical intercalation model results in unwinding of the DNA helix, which would lead to an increase in viscosity. In contrast, a partial and/or nonclassical intercalation of ligand could bend (or kink) the DNA helix, reduce its effective length and concomitantly, its viscosity [30]. Effect of the complexes on the viscosity of rod-like DNA is shown in Figure 6. The viscosity of DNA is not increased with the increase of the concentration of complexes, in contrast to that of proven DNA intercalator EtBr (= ethidium bromide). Based on the viscosity results, it was observed that these complexes bind with DNA through groove binding, result from DNA melting experiment further supported the above result.

### 4.4. DNA melting studies

**Figure 7:**
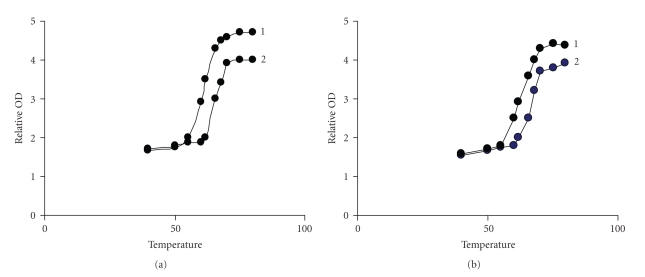
Plots of A/A_0_ versus temperature for the melting of CT DNA: (a) 1 only DNA spectra 2 DNA + complex 1, (b) 1 only DNA 2 DNA + complex 2.

As intercalation of the complexes into DNA base pairs causes stabilization of base stacking and hence raises the melting temperature of the double-stranded DNA, the DNA melting experiment is useful in establishing the extent of intercalation [34]. The complexes were incubated with CT DNA and their temperature raised from 25 to 85° and the absorbance at 260 nm was monitored. Conductivity and pH measurements were also carried out before and after heating the complexes to 85° through 1 hour [35]. The presence of monophasic melting curves with no change in pH. $\Delta T_{m}$ values of the DNA in presence of complexes is shown in Table 1, revealing avid DNA binding [36]. The complexes show $\Delta T_{m}$ values of 3° which is characteristic of a nonintercalative binding behavior (see Figure 7). viscosity experiments further support the nonintercalative binding.

### 4.5. Photocleavage of pBR322 DNA by Co(III) complexes

**Figure 8:**
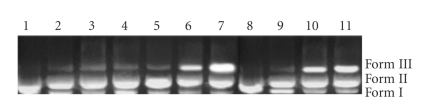
Photocleavage of pBR 322 DNA: lane 1 control plasmid DNA (untreated pBR 322), lanes 2–11 addition of complex (1) in amounts of 5, 10, 20, 30…*μ*l. Line 8 at 0 time lanes 7–10, + 5 *μ*m complex up on irradiation ($\lambda_{\text{irrd}}=360$ nm) at 5 minutes, 10 minutes, 20 minutes, 30 minutes.

There has been considerable interest in DNA endonucleolytic cleavage reactions which are activated by metal ions [37]. The delivery of high concentrations of metal ion to the helix, in locally generating oxygen or hydroxide radicals, yields an efficient DNA cleavage reaction. DNA photocleavage was monitored by relation of supercoiled circular pBR 322 (form I) into nicked circular (form II) and linear (form III). When circular plasimd DNA is subjected to electrophoresis, relatively fast migration will be observed for the supercoiled form (form I). If scission occurs on one strand (nicking), the supercoils will relax to generate a slower-moving open circular form (form II) [38]. If both strands are cleaved, a linear form (III) will be generated that migrates between forms I and II. Figure 8 shows the gel electrophoretic separations of plasmid pBR 322 DNA after incubation and irradiation at 360 nm with complex 1. This is the result of single stranded photocleavage of pBR322 DNA. That incubation with Co(III) without light yields significant strand scission. It is most likely that the reduction of Co(III) is the important step leading to DNA cleavage. Further study required to find out the path of reaction mechanism.

### 4.6. Anticancer studies

**Figure 9:**
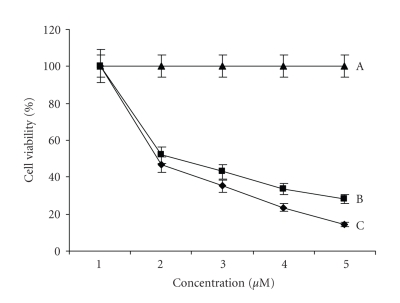
Effects of complex 2 [C], 1[B], and control [A] on the viability of CHO cells (human hepatocellular), following continuous incubation for 72 hours, with increasing drug concentration (0.1–500 *μ*M). Bars indicate standard error of the mean (SEM) and results were statistically significant from control at P<.05. Results are representative of three independent experiments (n=3).

**Figure 10:**
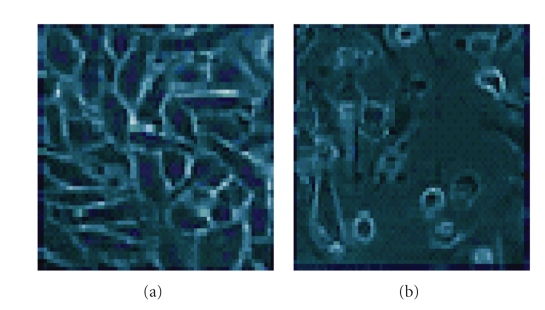
The morphological effects exerted by complexes on CHO cells 24 hours after treatment. Photographs were taken using a Nikon inverted light microscope (20X objective). (a) shows the untreated cells and while (b) shows cells treated with 0.5 mM of complex 2.

The ability of the cobalt complexes 1 and 2 to kill human-derived cancer cells was investigated using CHO cells and a standard bioassay, MTT. Cells were continuously exposed to test agent for 72 hours, and their effects on cellular viability was evaluated. It was intended that the results from these studies would allow the identification of those derivatives with cancer chemotherapeutic potential. Therefore, profiles of cell viability against complex concentration were established Figure 9 and were used to calculate the IC_50_ values for each derivative (see Table 2). Comparison of IC_50_ values allowed the relative potency of each of the test complexes to be determined and ranked. Photographs of treated and untreated CHO cells are presented in Figure 10. Both complexes screened displayed a concentration dependent cytotoxic profile. The order of the observed cytotoxicity was seen as complex 2 appearing as the potent.

## 5. CONCLUSIONS

In this study, we have attempted to unravel the DNA interaction of ethylenediamine pyridine Co(III) complexes. The binding behavior of complexes with DNA was characterized by absorption titration, fluorescence, and fluorescence quenching and viscosity measurements. The experimental results indicate that the complexes can bind to DNA through groove and Co(III) complex can efficiently cleave the plasmid pBR322. Overall, the results described explain to the DNA-binding, cleavage ability. The complex containing py ligand shows better anticancer activity than mepy. The efficiency of these complexes on various cancerous cell lines is presently being studied in our laboratory.

## Figures and Tables

**Table 1:** $\Delta T_{\text{m}}$ values of the DNA and complex + DNA.

Compound	${\text{T}}_{\text{M}}{}^{\circ }$C
CT DNA	60
[Co(en)_2_(py)_2_]^3+^	63
[Co(en)_2_(mepy)_2_]^3+^	63

**Table 2:** IC_50_ values of complexes 1, 2.

Complexes	IC_50_(nm) Mean ± SEM
[Co(en)_2_(mepy)_2_]Br_3_	1.8 *μ*M
[Co(en)_2_(py)_2_]Br_3_	1.75 *μ*M

## ABBREVIATIONS

CT DNA: Calf thymus DNA
py: Pyridine
mepy: Methylpyridine
en: ethylenediamine
EtBr: Ethedium bromide
